# Efficacy and Safety of a Plasma Vaginal Cleanser (WOMEN CARE^®^) Using Plasma-Activated Water in Suspected Vaginitis: A Multicenter Randomized Clinical Trial

**DOI:** 10.3390/biomedicines13123076

**Published:** 2025-12-12

**Authors:** Hye-Jin Cho, Min-Kyeong Kim, Yun-Seo Choe, Seo-Yeon Son, Chi-Gu Kang, So-Jung Lim, Sooyong Kim, Hoonseong Choi, Un Suk Jung, Ju-Seop Kang

**Affiliations:** 1 Honest Women’s Clinic, Seoul 07333, Republic of Korea; pamina73@hanmail.net (H.-J.C.);; 2 ON Women’s Clinic, Seoul 04571, Republic of Korea; onwclinic7@kakao.com; 3 Department of Pharmacology, College of Medicine, Hanyang University, Seoul 04763, Republic of Korea; ilovekcg@hanyang.ac.kr (C.-G.K.); elly0216@hanyang.ac.kr (S.-J.L.); 4 Corporate Research Institute, 4Hglobal, Bucheon 14559, Republic of Korea; sooyong.t.kim@gmail.com (S.K.); telcontar@naver.com (H.C.); 5 Department of Obstetrics and Gynecology, Hanyang University Guri Hospital, Hanyang University College of Medicine, Guri 11923, Republic of Korea; petrow@hanyang.ac.kr

**Keywords:** vaginitis, plasma-activated water (PAW), WOMEN CARE^®^, vaginal microbiota, Nugent score, non-antibiotic therapy

## Abstract

**Background/Objectives**: Vaginitis is a prevalent inflammatory disorder of the vaginal mucosa, frequently arising from its anatomical proximity to the anorectal region and a microenvironment conducive to pathogen colonization and dysbiosis. This prospective, multicenter, randomized, third-party-blinded study assessed the efficacy and safety of a plasma vaginal cleanser (WOMEN CARE^®^) employing plasma-activated water (PAW) as a non-pharmacological alternative to conventional antimicrobials for restoring vaginal homeostasis. **Methods**: Women aged ≥19 years with clinically suspected vaginitis were assigned to either the experimental group (WOMEN CARE^®^) or the control group (standard pharmacotherapy). The primary endpoint was the proportion of participants exhibiting decreased Nugent scores between baseline and Visit 4. **Results**: Of 144 participants in the experimental group, 125 completed the study. The experimental group showed comparable outcomes to standard pharmacotherapy group across Nugent scores, vaginal pH, and symptoms severity, with pathogen suppression confirmed as non-inferior. Additionally, PAW exerted anti-HPV activity through a potential effect against new genotypic HPV infection. While the control group experienced antibiotic-associated adverse effects (e.g., headache, abdominal discomfort, nausea), no treatment-related adverse events occurred in the WOMEN CARE^®^ group. **Conclusions**: These results indicate that PAW vaginal cleansing provides an effective, safe, non-antibiotic approach for managing vaginitis and maintaining vaginal ecological balance.

## 1. Introduction

The human microbiota, consisting of bacteria, viruses, fungi, and protozoa, is distributed throughout the gastrointestinal, respiratory, reproductive, oral, and integumentary systems. Within this context, the female reproductive tract is particularly vulnerable to infection due to its anatomical proximity to the urethra and anus and its warm, moist environment that promotes microbial colonization and overgrowth. Vulvovaginal infections, collectively referred to as vaginitis, are primarily caused by bacteria, fungi, or protozoa. The most prevalent forms include bacterial vaginosis (BV), vulvovaginal candidiasis (VVC), and trichomoniasis, accounting for approximately 22–50%, 17–39%, and 4–39% of cases, respectively [1].

Current therapeutic approaches rely on oral or intravaginal administration of antibiotics or antifungal agents, depending on the etiology. However, recurrence remains a significant clinical challenge, with both bacterial vaginosis (BV) and vulvovaginal candidiasis (VVC) showing recurrence rates of approximately 60% within one year [2,3], largely attributable to persistent biofilms and antifungal resistance. These limitations have prompted exploration of alternative strategies such as probiotics, boric acid suppositories, herbal extracts, and ozone-based cleansing solutions.

In recent years, plasma-based medical technologies have emerged as promising antimicrobial modalities. WOMEN CARE^®^ is a vaginal cleansing device that generates plasma-activated water (PAW), a solution enriched with reactive oxygen and nitrogen species formed through plasma–water interactions [4,5]. PAW exhibits broad-spectrum antimicrobial activity against bacteria, fungi, and viruses, disrupts and removes biofilms, and, importantly, does not promote microbial resistance [4,6,7]. Delivered directly into the vaginal rugae, PAW facilitates mechanical and biochemical removal of pathogens and biofilms, representing a novel non-pharmacological alternative to conventional antimicrobial therapy.

The present study was designed to evaluate the efficacy and safety of PAW delivered via the WOMEN CARE^®^ device in women with suspected vaginitis. Outcomes include Nugent scoring of Gram-stained vaginal smears, PCR-based pathogen detection, vaginal pH, symptom severity, clinical improvement, and patient satisfaction. Collectively, these findings are expected to provide comprehensive evidence on the potential of PAW to restore vaginal health and maintain microbial homeostasis.

## 2. Materials and Methods

### 2.1. Study Design

This study was conducted as a prospective, multicenter, randomized, comparative clinical trial with third-party blinding. The objective was to evaluate the efficacy and safety of WOMEN CARE^®^ in improving vaginitis and the vaginal environment among patients with suspected vaginitis.

Between July 2024 and April 2025, the participants were recruited from Honest Women’s Clinic and ON Women’s Clinic. The study complied with guidelines of the Declaration of Helsinki and was approved by the Institutional Review Board of Korea National Institute for Bioethics Policy (P01-202407-06-001, 19 July 2024), and was registered at CRIS registry (KCT0009731, 28 August 2024). Eligible women aged 19 years or older with suspected vaginitis were randomly assigned to either the experimental group (WOMEN CARE^®^) or the control group (standard pharmacological therapy). Clinical outcomes were assessed by comparing pre- and post-treatment findings, including polymerase chain reaction (PCR) assays for 12 sexually transmitted pathogens, Gram staining and microbial culture results, and symptom evaluation scores. The primary endpoint of the study was the proportion of participants with reduced Nugent score, which reflects the severity of pathogen-associated vaginitis. Non-inferiority of the cleansing effect of WOMEN CARE^®^ was evaluated based on this Nugent score change relative to the control group.

### 2.2. In Vitro HPV-Infected Cell Viability Test

#### 2.2.1. Preparation PAW and Cell Treatment

Plasma activation was performed using a PAW-generating device (SJ Global, Bucheon, Republic of Korea) with a fixed treatment time of 40 min. Following plasma activation, the incubation times were set at 0, 5, 10, 20, 30, and 40 min. From a 3 L water container, PAW was collected according to the stopcock position (top, middle, and bottom layers), with 5 mL transferred into 50 mL conical tubes (SPL, 50050) from each layer. Cells seeded in 96-well plates were treated with PAW under varying conditions, including retention time, stopcock layer, and treatment volume (20 µL PAW + 80 µL medium). Tap water was used as the control under the same conditions.

#### 2.2.2. HPV-Infected Cell Viability Assay

HPV-infected cells (KCBL-21550-cervix, uterine) were purchased from a Korea Cell Line Bank (Seoul, South Korea). Cell viability was evaluated at 48 h after treatment using the MTT assay. Thiazolyl Blue Tetrazolium Bromide (MTT; SIGMA-ALDRICH, St. Louis, MO, USA, M5655-100MG) was dissolved in PBS (WELGENE, Gyeongsan, Republic of Korea, Cat. LB004-02) at a concentration of 5 mg/mL. A 10 µL aliquot of MTT solution was added to each well, and after 3 h of incubation, the formation of formazan crystals was confirmed. The medium was then removed by suction, and 100 µL of dimethyl sulfoxide (DMSO; SIGMA-ALDRICH, D4540-500ML) was added to dissolve the crystals. Absorbance was measured at 570 nm using a microplate reader (VERSAmax, Tulsa, OK, USA, Molecular Devices).

### 2.3. Clinical Study Population

Eligible participants were women aged 19 years or older with suspected vaginitis who voluntarily provided written informed consent after receiving a detailed explanation of the study procedures. Participants were also required to demonstrate willingness and ability to comply with the requirements of the study protocol.

Exclusion criteria were as follows: a history of hypersensitivity or adverse reactions to standard therapeutic agents; current use of an intrauterine device; pregnancy; intellectual or psychiatric conditions that could impair autonomous decision-making; absence of prior sexual experience; administration of antibiotics or antifungal agents within one week prior to enrollment; and any other condition deemed by the investigator to make participation inappropriate.

The required sample size for the non-inferiority evaluation was calculated assuming an expected response rate of 73.38% in the control group (based on a previous meta-analysis [8]) and a target response rate of 67% in the experimental group. Although previous studies [4,6,7] assessed different endpoints, p_E_ was conservatively set at approximately 90% of the control rate to ensure adequate power. A non-inferiority margin of Δ = 0.10 (10%) was prespecified, representing the maximum clinically acceptable difference and considered appropriate by the study investigators. Using a one-sided α = 0.025 and 80% power, the required sample size per group was calculated using the standard normal approximation for two independent proportions. Allowing for a 15% dropout rate, the target sample size per group was set at 144 participants, resulting in a total enrollment target of 288 participants.

$$n=\frac{{\left(Z_{1-\alpha }+Z_{1-\beta }\right)}^{2}\left[p_{C}\left(1-p_{C}\right)+p_{E}\left(1-p_{E}\right)\right]}{{\left(p_{C}-p_{E}+∆\right)}^{2}}\approx 122$$

**Figure 1:**
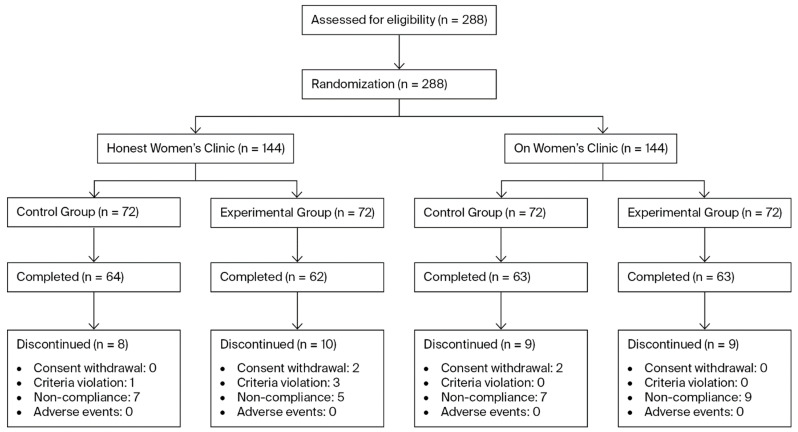
Flow diagram of patients assigned to the study.

To account for this, the final target was determined to be 72 participants per group per site, resulting in a total enrollment target of 288 participants across both sites. A total of 288 participants were enrolled (144 subjects in the control group and 144 subjects in the experimental group). Of these, 36 participants prematurely discontinued the study. The Reasons for discontinuation included withdrawal of informed consent (*n* = 4), violation of inclusion or exclusion criteria (*n* = 4), and noncompliance with investigator instructions, such as failure to adhere to scheduled visits (*n* = 28) (Figure 1).

### 2.4. Study Methods and Randomization

This prospective, multi-center, randomized, third-party blinded, comparative clinical trial evaluated the efficacy and safety of plasma-activated water produced by the vaginal plasma cleanser (WOMEN CARE^®^) for eliminating vaginitis-associated pathogens (bacteria, fungi, viruses) and improving the vaginal environment.

Participants were randomly assigned to either the experimental group or the control group. Sequentially numbered, opaque, sealed envelopes were used for simple randomization without restriction. The randomization sequence was generated by an independent statistician. Sealed envelopes were prepared by a clinical research associate (CRA) who was not involved in participant enrolment or assignment. These envelopes were opened by site investigators only after participant consent and completion of baseline assessments. Those allocated to the experimental group received treatment with WOMEN CARE^®^, while participants in the control group were provided with standard pharmacological therapy according to the diagnosed condition. Randomization was performed to ensure comparability between groups and to minimize selection bias.

**Figure 2:**
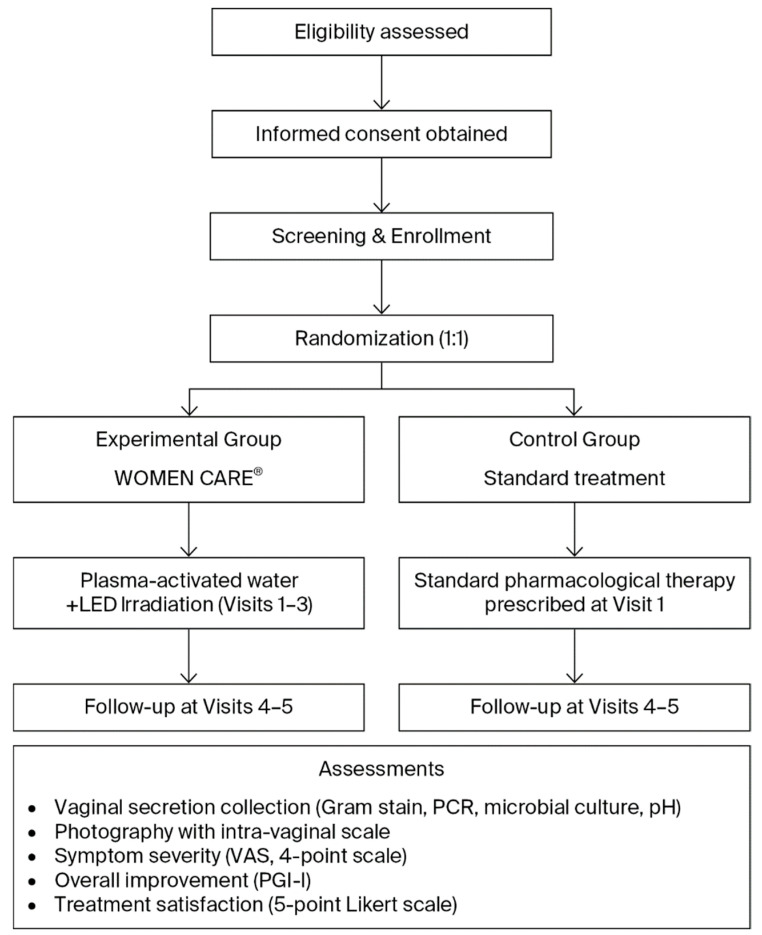
Flowchart of Study Design and Procedure.

Patients with clinically confirmed or suspected vaginitis were assessed before and after treatment using Gram staining, PCR assays for 12 sexually transmitted infections and HPV, vaginal pH measurement, photographic documentation, and patient satisfaction surveys (Table 1, Figure 2). Participants were randomized in a 1:1 ratio into two groups. And outcomes were compared between the WOMEN CARE^®^ and standard pharmacological treatment groups. At the two institutions, Honest Women’s Clinic and ON Women’s Clinic, eligible participants meeting the inclusion and exclusion criteria were enrolled in the study following individual screening and randomly assigned to either the experimental group or the control group. Within each institution, sealed envelopes numbered 1 through 144 were randomly selected to assign participants to the experimental or control group, ensuring an equal 1:1 allocation probability.

### 2.5. Intervention

#### 2.5.1. Experimental and Control Group

In the experimental group, PAW was generated using the WOMEN CARE^®^ device. Using the Clean & Suction system, approximately 150–200 mL was sprayed and suctioned for 1–2 min to cleanse vaginal pathogens, followed by 30 s of intra-vaginal LED irradiation. Participants in the experimental group received this procedure at Visit 1, Visit 2, and Visit 3. In the control group, standard pharmacological treatments were administered at Visit 1 according to the diagnosed condition. For bacterial vaginosis, oral metronidazole 500 mg was prescribed for 7 consecutive days. Vulvovaginal candidiasis was treated with a single oral dose of fluconazole 150 mg. Infections caused by Ureaplasma urealyticum were managed with either doxycycline 100 mg administered orally twice daily for 7 days or a single oral dose of azithromycin 1 g. Human papillomavirus (HPV) infection was managed expectantly, as spontaneous resolution was generally anticipated and no active pharmacological intervention was required. Throughout the trial, no concomitant treatment or care differing between the experimental and control groups was administered.

#### 2.5.2. Participant and Informed Consent Collection

Prior to the initiation of any screening procedures, written informed consent was obtained from all participants who met the inclusion and exclusion criteria. The principal investigator and co-investigators provided a detailed explanation of the study, using the participant information sheet and consent form as reference. Participants were given sufficient time to understand all foreseeable risks, benefits, and study procedures before providing consent. A copy of the signed consent form was provided to the participant, while the original was securely retained by the investigator. Investigators maintained a comprehensive record of all participants who consented to participate. Participants were informed that their study-related data might be used by the investigators in accordance with applicable regulations and that medical records might be reviewed by clinical research monitors, auditors, institutional review boards, and regulatory authorities.

In the event of any amendments to the study protocol, the participant information sheet and consent form were revised to reflect such changes. All revisions were reviewed and approved by the institutional review board. Both newly enrolled participants and those already participating were informed of any changes and asked to provide signatures on the updated consent forms. Participants were promptly notified of any new information that might influence their decision to continue in the study, and investigators discussed the continuation of participation with them. Informed consent was obtained by the principal investigator or designated co-investigators at the Honest Women’s Clinic and ON Women’s Clinic. The consent process was conducted among patients who met all inclusion and exclusion criteria at the time of their clinic visit, prior to enrollment in the study.

### 2.6. Statistical Analysis

All data obtained from study participants were analyzed according to three populations: the Safety population, the Full Analysis Set (FAS), and the Per-Protocol (PP) population. The PP population served as the primary efficacy analysis set to demonstrate non-inferiority compared to standard treatment, while supplementary analysis was performed in the FAS. Safety analysis was conducted in the Safety population. In cases where results differ among populations, findings from each analysis were reported with explanation (Table 2). Complete-case analyses were performed, excluding participants with missing outcome data from the corresponding analyses. Continuous variables were summarized as means ± standard deviations and analyzed using a two-sample Student’s *t*-test or Wilcoxon’s rank-sum test if the normality assumption is violated. Categorical variables were summarized as frequencies and percentages and analyzed using the chi-square test or Fisher’s exact test when more than 25% of cells have expected counts below five. The significance level was set at α = 0.05 for all tests.

Adverse events (AEs) were classified according to MedDRA and summarized by System Organ Class (SOC) and Preferred Term (PT). The frequency (number and percentage) of AEs, medical device-related adverse reactions, and serious adverse events (SAEs) were reported, along with summaries by severity and causality. Participants who experienced serious adverse reactions or withdraw prematurely due to adverse reactions were listed individually. When necessary, statistical significance of differences in overall AE incidence between groups was assessed using the chi-square test or Fisher’s exact test.

All statistical analyses were performed using software R, version 4.4.2; R Foundation for Statistical Computing, Vienna, Austria, and RStudio, version 2025.5.1.513. Descriptive statistics (number of observations, mean, standard deviation, median, minimum, and maximum) were reported for relevant endpoints. Between-group differences were analyzed using ANCOVA where appropriate, adjusting for baseline values.

## 3. Results

Among the 288 participants enrolled in this clinical study, 36 individuals who discontinued prematurely were excluded, resulting in a Full Analysis Set (FAS) of 252 participants (control group: 127; experimental group: 125). For efficacy analyses, the Per-Protocol (PP) population was defined by excluding participants who were STD PCR-negative at baseline, comprising 109 participants in the control group and 102 participants in the experimental group, which constituted the primary analysis set. Although exclusion of PCR-negative participants was not explicitly prespecified in the protocol, this criterion was applied to ensure that efficacy was assessed only among participants with confirmed pathogen positivity at baseline, consistent with the study objective of evaluating treatment effects on pathogen-associated vaginitis. It should be noted that, even within the PP population, the number of participants included in some secondary efficacy analyses differed from the primary analysis set. This was due to missing measurements or incomplete responses to patient-reported questionnaires, and these variations reflect data availability rather than post-randomization exclusions.

There were no differences in the basic demographic characteristics of the participants (Table 3). A notable age disparity was found between the control and experimental groups; however, this factor did not affect any of the other results.

Participants assigned to the control group received standard pharmacological treatment based on clinical symptoms and laboratory results. Participants randomized to the experimental group received vaginal cleansing using plasma-activated water (PAW) from the WOMEN CARE^®^ medical device across three sessions (Visits 1, 2, and 3).

To evaluate the effects of the interventions on vaginitis and vaginal environment improvement, microbiological assessments and symptoms questionnaires were conducted before and after treatment in both groups. Key outcome measures, including Nugent score, PCR results, vaginal pH, and symptom scores, were compared and analyzed between the control and experimental groups.

Changes in the vaginal microbiota were assessed using Nugent scores obtained from Gram-stained vaginal smear samples. The results from specimens collected at Visit 1 (pre-treatment baseline) and Visit 4 were compared. The proportions of participants whose Nugent scores decreased before and after treatment were 19.27% (21/109) in the control group and 30.39% (31/102) in the experimental group (Table 4). The risk difference (experimental − control) was 0.111 (95% CI: −0.005 to 0.227), with a prespecified non-inferiority margin of Δ = 0.10. As the lower bound of the CI exceeded −Δ, the PAW intervention met the non-inferiority criterion relative to standard treatment. Fisher’s exact test yielded a *p*-value of 0.078, indicating no statistically significant difference between groups (Table 4). When comparing Visit 5 with baseline, the proportions of participants with decreased Nugent scores were also comparable, at 21.10% (23/109) in the control group and 31.37% (32/102) in the experimental group (*p* = 0.1163).

To provide a more clinically meaningful comparison, participants with a baseline Nugent score of 0 were excluded from a secondary analysis, as these participants could not show a reduction in score. In this subset, the proportion of participants with decreased Nugent scores was 67.7% (21/31) in the control group and 68.9% (31/45) in the experimental group (Table 5). Furthermore, comparisons of the rates of change in Nugent scores between the two groups showed no statistically significant differences at either Visit 4 or Visit 5 relative to baseline (Table 6).

After excluding participants with a baseline Nugent score of 0, no significant differences were observed between the control group (*n* = 31) and the experimental group (*n* = 45) at Visit 1, and this trend persisted at Visit 4 and Visit 5. At baseline (Visit 1), the mean Nugent scores were 2.259 ± 2.352 in the control group and 1.756 ± 1.335 in the experimental group, with no statistically significant difference (*p* = 0.5343). Similarly, no significant intergroup differences were identified at Visit 4 (0.775 ± 1.522 vs. 0.889 ± 1.266, *p* = 0.279) or Visit 5 (0.904 ± 1.641 vs. 0.845 ± 1.461, *p* = 0.3114). These findings indicate that there were no statistically significant differences in Nugent scores between the two groups across the study period.

Among participants with baseline vaginal pH > 5 (control, *n* = 67; experimental, *n* = 63), mean vaginal pH did not differ significantly between groups at any visit. At Visit 1, pH was 6.478 ± 0.705 (control) and 6.556 ± 0.714 (experimental, *p* = 0.4657); at Visit 4, 6.015 ± 1.052 and 5.921 ± 1.112 (*p* = 0.4541); and at Visit 5, 5.792 ± 0.914 and 5.810 ± 0.998 (*p* = 0.842). The proportion of participants showing a pH decrease from Visit 1 was 47.76% vs. 61.90% at Visit 4, and 62.69% vs. 66.67% at Visit 5 (control vs. experimental), with no significant differences between groups (Table 7).

The PCR analysis identified 12 STI pathogens. In the control group, six types were consistently detected both Visit 1 and 5. In the experimental group, eight types were identified at baseline, but CT and HSV-2 were no longer detected Visit 5, resulting in a reduction to six types. Overall, only minimal reduction was observed, which may be attributed to continuous exposure to infection associated with regular sexual activity. (Table 8)

Semi-quantitative changes in 12 STI pathogens were assessed by PCR at Visit 1 (pre-treatment) and Visit 5(after-treatment). Among participants positive for at least one pathogen (control, *n* = 103; experimental, *n* = 98), the proportion showing a reduction was: Ureaplasma parvum, 12.96% vs. 14.58% (*p* = 1); Ureaplasma urealyticum, 50% vs. 37.5% (*p* = 0.5525); Mycoplasma hominis, 28.57% vs. 30.77% (*p* = 1); Candida albicans, 55.36% vs. 36.73% (*p* = 0.0776); and Gardnerella vaginalis, 24% vs. 17.14% (*p* = 0.4123). No statistically significant differences were observed between groups (Table 9).

To assess the severity of participants’ symptoms, six items (dryness, itching, burning, changes in vaginal discharge, dysuria, and dyspareunia) were evaluated using a 4-point scale (absent, mild, moderate, severe). In addition, a 10 cm visual analog scale (VAS) was used to assess overall discomfort, including other symptoms. Participants with a total symptom score of 0 or missing values at the first visit were excluded, and the sum of symptom scores and 10 cm VAS values were compared. Compared with pre-treatment, both the control and experimental groups showed decreases in symptom scores and VAS values after treatment. However, differences between groups were small relative to individual variability, and no statistically significant differences were observed (Table 10 and Table 11).

Participant-perceived symptom improvement and satisfaction with the treatment were assessed using a 5-point Likert scale at Visits 4 and 5. Comparisons between groups showed no significant differences in either improvement or satisfaction (Table 12).

At baseline, 50 patients in the control group and 41 patients in the experimental group tested positive for HPV PCR test (Table S2 and S3). After intervention, HPV was detected in 17 patients in the control group and 8 patients in the experimental group. Among them, HPV was no longer detectable in 21 patients in the control group and 20 patients in the experimental group (Table 13 and Table S1). No statistically significant differences were observed between the groups. However, among subjects with newly acquired genotypes after treatment, the number of newly acquired genotypes was higher in the control group (17/11 = 1.545) than in the experimental group (8/8 = 1.0), with a statistically significant difference (Wilcoxon rank sum test, *p* = 0.03639). The incidence of newly detected HPV genotypes was lower in the experimental group than in the standard drug therapy group, indicating a potential preventive effect of PAW against HPV infection.

No significant differences were observed among PAW samples obtained from the up-per, middle, and lower layers. The PAW with a 20:80 ratio exhibited the highest efficacy. Treatment with 20% PAW medium for 48 h resulted in an approximately 10% decrease in cell viability, indicating that PAW reduces the viability of HPV-infected CaSki cells (Figure S1).

## 4. Discussion

The female reproductive tract is inherently vulnerable to infection due to its anatomical proximity to the urethra and anus, frequent exposure to pathogens, and the presence of a warm and moist environment that supports microbial growth. Consequently, vulvovaginal infections, including bacterial vaginosis, vulvovaginal candidiasis, and trichomoniasis, remain prevalent [9].

Despite the availability of standard pharmacological treatments, recurrence rates, particularly for vulvovaginal candidiasis, remain high [10,11]. This is largely attributed to biofilm formation, which protects pathogens from antimicrobial agents and facilitates persistent infection [12]. Biofilm-associated resistance highlights the urgent need for alternative therapeutic approaches that can disrupt microbial communities more effectively than conventional antibiotics or antifungal drugs [13].

Cold atmospheric plasma (CAP) and plasma-activated water (PAW) have emerged as promising candidates in this regard [14]. PAW exerts antimicrobial effects through the generation of reactive oxygen and nitrogen species, which can disrupt biofilms and eliminate bacteria, fungi, and viruses. Importantly, PAW does not induce microbial resistance, making it an attractive non-pharmacological alternative for recurrent or resistant infections [15,16].

Beyond antibacterial and antifungal properties, both CAP and PAW have demonstrated antiviral effects through the generation of reactive oxygen and nitrogen species, which damage viral DNA, RNA, and proteins. Previous studies have shown efficient inactivation of bacteriophages under various plasma conditions, although the precise molecular mechanisms remain unclear and appear to depend on environmental factors and viral structure [17,18,19,20].

Building on this evidence, the present study investigated the effects of PAW on HPV-infected cell lines (CaSki). Incubation with 20% PAW medium for 48 h resulted in an approximate 10% reduction in cell survival, suggesting that PAW not only inactivates viral particles but also impairs the viability of HPV-infected host cells. These in vitro findings are consistent with clinical observations, where the experimental group receiving PAW treatment demonstrated a reduction in HPV genotypes compared with the control group. Taken together, these results indicate that PAW may exert anti-HPV activity through a dual mechanism involving both direct viral inactivation and decreased survival of infected cells.

The WOMEN CARE^®^ device, which generates PAW for vaginal cleansing, was specifically designed to translate this principle into a clinical application. Previous studies have reported reductions in pathogens such as Mycoplasma hominis, Ureaplasma species, and Candida albicans without adverse effects, supporting both the safety and potential efficacy of this device [4,5,6,7].

The present clinical trial further extends these findings by directly comparing PAW therapy with standard pharmacological treatment in women with suspected vaginitis. Across key clinical indicators—including Nugent score, vaginal pH, symptom scores, and pathogen detection—PAW demonstrated non-inferiority to conventional pharmacological therapies. Especially, the incidence of newly detected HPV genotypes was lower in the PAW treatment experimental group than in the conventional pharmacological control group, indicating a potential preventive effect of PAW against HPV infection.

The etiology of Candida albicans infection is multifactorial, encompassing a variety of environmental and physiological factors that are difficult to control, such as prolonged moisture exposure, stress, and conditions affecting host microbiota. In the present study, PAW treatment demonstrated efficacy comparable to standard therapy. The semi-quantitative reduction in C. albicans was slightly lower in the experimental group compared to the control group (36.73% vs. 55.36%); however, this difference was not statistically significant (*p* = 0.07756), and analysis of Nugent score changes also revealed no meaningful differences between groups. Among participants who were PCR-positive for C. albicans at baseline—a subgroup not reported in the main results—16.7% of the control group (*n* = 54) and 30.6% of the experimental group (*n* = 49) showed a decrease in Nugent score from baseline to Visit 4. When restricting to participants with non-zero baseline Nugent scores (13 control, 23 experimental), the proportions showing a reduction were 69.2% and 65.2%, respectively. Taken together, these results indicate no significant difference between the two groups.

Notably, no adverse events were reported in the experimental group, in contrast to the known side effects commonly associated with antibiotics and antifungal agents. In the control group, two participants reported headache, while abdominal pain and nausea were reported by one participant each. These results suggest that PAW therapy not only achieves comparable clinical outcomes but also provides a favorable safety profile.

Overall, these findings support the use of PAW delivered via WOMEN CARE^®^ as a safe and effective alternative to standard pharmacological therapy for vaginitis. By offering a non-antibiotic, non-antifungal strategy that avoids the limitations of drug resistance and side effects, PAW may represent a valuable addition to the management of vaginitis and the maintenance of vaginal health.

Since the subjects returned to their daily lives after the intervention, no restrictions were placed on their daily lives. Therefore, various lifestyle factors (including sexual life) may act as confounding variables. The inability to properly control for these con-founding variables is also a limitation of this study.

## Figures and Tables

**Table 1:** Study Schedule and Assessments.

Visit	Experimental Group	Control Group	Assessments
1	WOMEN CARE**^®^** procedure	Standard Pharmacological Prescription	Vaginal secretion collection, PCR, Gram-stain, pH, photography, symptom survey (VAS)
2	WOMEN CARE**^®^** procedure	-	Vaginal secretion collection, photography, symptom survey (VAS)
3	WOMEN CARE**^®^** procedure	-	Vaginal secretion collection, photography, symptom survey (VAS)
4	Follow-up	Follow-up	Vaginal secretion collection, PCR, Gram-stain, pH, photography, symptom survey (VAS), PGI-I, satisfaction
5	Follow-up	Follow-up	Vaginal secretion collection, PCR, Gram-stain, pH, photography, symptom survey (VAS), PGI-I, satisfaction

Visit 1 (Day 0, post-randomization); Visit 2 (Days 1–4); Visit 3 (Days 2–7); Visit 4 (Days 7–14); Visit 5 (Days 28–38). Safety outcomes were monitored continuously throughout the study period.

**Table 2:** Data Analysis for Efficacy Endpoints.

Endpoint	Outcome Measure	Population	Statistical Method
1st efficacy	Proportion of participants with reduced Nugent score (baseline–Visit 4)	PP	Chi-square or Fisher’s exact test
2nd efficacy	Proportion of participants with decreased STI pathogen detection (baseline–Visit 5, PCR)	PP/FAS	Chi-square or Fisher’s exact test
	Change in Nugent score (Visit 1–Visit 4)	PP/FAS	Descriptive statistics; *t*-test between groups
	Proportion of participants with decreased Nugent score (baseline–Visit 5)	PP/FAS	Chi-square or Fisher’s exact test
	Proportion of participants with improved vaginal pH (Visit 1–Visits 4 and 5)	PP/FAS	Chi-square or Fisher’s exact test
	Symptom questionnaire score changes (VAS, individual items)	PP/FAS	Two-way RM ANOVA
	Participant satisfaction (Visits 4 and 5, Likert scale)	PP/FAS	Descriptive statistics; ANCOVA for between-group comparison
	Overall symptom improvement (Likert scale)	PP/FAS	Descriptive statistics
	Nugent score, vaginal pH, symptom scores over time	Experimental group	One-way RM ANOVA; *t*-tests for individual time points

**Table 3:** Baseline Physical Characteristics of the Control and Experimental Groups (*p*-value; Wilcoxon Rank Sum Test).

	Mean ± SD		*p*-Value
	Control Group (*n* = 109)	Experimental Group (*n* = 102)	
Age (year)	32.27 ± 8.03	35.33 ± 9.77	0.0126
Height (cm)	162.85 ± 4.78	161.90 ± 5.19	0.2112
Weight (kg)	55.52 ± 7.34	55.47 ± 7.65	0.8586

**Table 4:** Proportion of participants with decreased Nugent scores at Visit 4/5 compared to Visit 1 (*p*-value; Fisher’s exact test).

Visit	Control Group (*n* = 109)	Experimental Group (*n* = 102)	*p*-Value
1–4	21/109 (19.27%)	31/102 (30.39%)	0.07841
1–5	23/109 (21.10%)	32/102 (31.37%)	0.1163

**Table 5:** Proportion of participants with decreased Nugent scores at Visit 4/5 compared to Visit 1 (excluding participants with Nugent score of 0 at Visit 1) (*p*-value; Fisher’s exact test).

Visit	Control Group (*n* = 31)	Experimental Group (*n* = 45)	*p*-Value
1–4	21/31 (67.74%)	31/45 (68.89%)	1
1–5	23/31 (74.19%)	32/45 (71.11%)	0.8005

**Table 6:** Reduction Rate of Nugent Scores (*p*-value; Wilcoxon Rank Sum Test).

Visit	Mean ± SD		*p*-Value
	Control Group (*n* = 31)	Experimental Group (*n* = 45)	
1–4	0.602 ± 0.537	0.392 ± 0.899	0.5650
1–5	0.519 ± 0.841	0.509 ± 0.730	0.7012

**Table 7:** Proportion of Participants with Decreased Vaginal pH at Visits 4/5 Compared with Visit 1 (*p*-value; Fisher’s Exact Test).

Visit	Mean ± SD		*p*-Value
	Control Group (*n* = 67)	Experimental Group (*n* = 63)	
1–4	32/67 (47.76%)	39/63 (61.90%)	0.2082
1–5	42/67 (62.69%)	42/63 (66.67%)	0.1604

**Table 8:** PCR Results for 12 STI Pathogens (Before: Visit 1, After: Visit 5).

Group		MH	MG	UU	UP	CT	GV	HSV2	CA
Control Group (*n* = 103)	Visit 1	14	0	22	54	0	75	2	56
	Visit 5	15	0	21	53	0	66	1	36
Experimental Group (*n* = 98)	Visit 1	13	1	24	48	1	70	4	49
	Visit 5	13	1	20	48	0	68	0	46

MH, *Mycoplasma hominis*; MG, *Mycoplasma genitalium*; UU, *Ureaplasma urealyticum*; UP, *Ureaplasma parvum*; CT, *Chlamydia trichomatis*; GV, *Gardnerella vaginalis*; HSV2, *Herpes simplex virus 2*; CA, *Candida albicans*.

**Table 9:** Proportion of Participants Showing Semi-quantitative Reduction in STI Pathogens after Intervention.

STI	MH	UU	UP	GV	CA
Control Group	4/14 (28.57%)	11/22 (50%)	7/54 (12.96%)	18/75 (24%)	31/56 (55.36%)
Experimental Group	4/13 (30.77%)	9/24 (37.5%)	7/48 (14.58%)	12/70 (17.14%)	18/49 (36.73%)
*p*-value	1	0.5525	1	0.4123	0.07756

STI, Sexual transmitted infection; MH, *Mycoplasma hominis*; UU, *Ureaplasma urealyticum*; UP, *Ureaplasma parvum*; GV, *Gardnerella vaginalis*; CA, *Candida albicans*.

**Table 10:** Symptoms Assessment Scores (*p*-value; Wilcoxon Rank Sum Test).

Visit	Mean ± SD		*p*-Value
	Control Group (*n* = 109)	Experimental Group (*n* = 100)	
1	6.771 ± 3.240	6.942 ± 3.374	0.7139
4	3.111 ± 2.492	3.344 ± 2.771	0.7221
5	3.166 ± 2.840	3.020 ± 3.090	0.4158

**Table 11:** 10 cm VAS Scores (*p*-value; Wilcoxon Rank Sum Test).

Visit	Mean ± SD		*p*-Value
	Control Group (*n* = 106)	Experimental Group (*n* = 102)	
1	5.119 ± 2.093	5.587 ± 2.058	0.3744
4	2.361 ± 1.926	2.816 ± 2.243	0.3059
5	2.367 ± 2.253	2.220 ± 2.180	0.5480

**Table 12:** Improvement and Satisfaction Scores (*p*-value; Wilcoxon Rank Sum Test).

	Visit	Control Group	Experimental Group	*p*-Value
PGI-I	4	3.981 ± 0.789	4.041 ± 0.728	0.5541
	5	4.020 ± 0.893	4.142 ± 0.686	0.4487
Satisfaction Index	4	4.049 ± 0.730	4.152 ± 0.706	0.2607
	5	4.068 ± 0.780	4.162 ± 0.725	0.3276

**Table 13:** Comparison of HPV genotypic changes after intervention.

Contents	Control Group (*n* = 50)	Experimental Group (*n* = 41)	*p*-Value
Incidence of new genotypes, *n* (%)	17 (34%)	8 (19.5%)	0.6121
Cases with newly detected genotype, *n* (%)	11 (22%)	8 (19.5%)	0.0364
Number of lost genotypes, *n* (%)	21 (42%)	20 (48.8%)	0.5102
Subjects with lost genotypes, *n* (%)	16 (32%)	16 (39%)	0.5151

## Data Availability

The original contributions presented in this study are included in the article/Supplementary Material. Further inquiries can be directed to the corresponding author.

## Supplementary Materials

The following supporting information can be downloaded at https://www.mdpi.com/article/10.3390/biomedicines13123076/s1: Figure S1 and Table S1: Effect of PAW on HPV-infected cell line (viability test); Table S2: Change in HPV Genotype status after Intervention in Control Group; Table S3: Change in HPV Genotype status after Intervention in experimental Group.
