# Supplementary material for: Efficacy and Safety of a Plasma Vaginal Cleanser (WOMEN CARE®) Using Plasma-Activated Water in Suspected Vaginitis: A Multicenter Randomized Clinical Trial
Source: Biomedicines. 2025 Dec 12;13(12):3076. doi: 10.3390/biomedicines13123076 (PMC12730254; doi:10.3390/biomedicines13123076)
Supplement: Supplementary file 1 [file biomedicines-13-03076-s001.zip › Supplement 1_Figure and Table(cell viability%).pdf]

Figure S1 and Table S1. Effect of PAW on HPV-infected cell line (viability test)

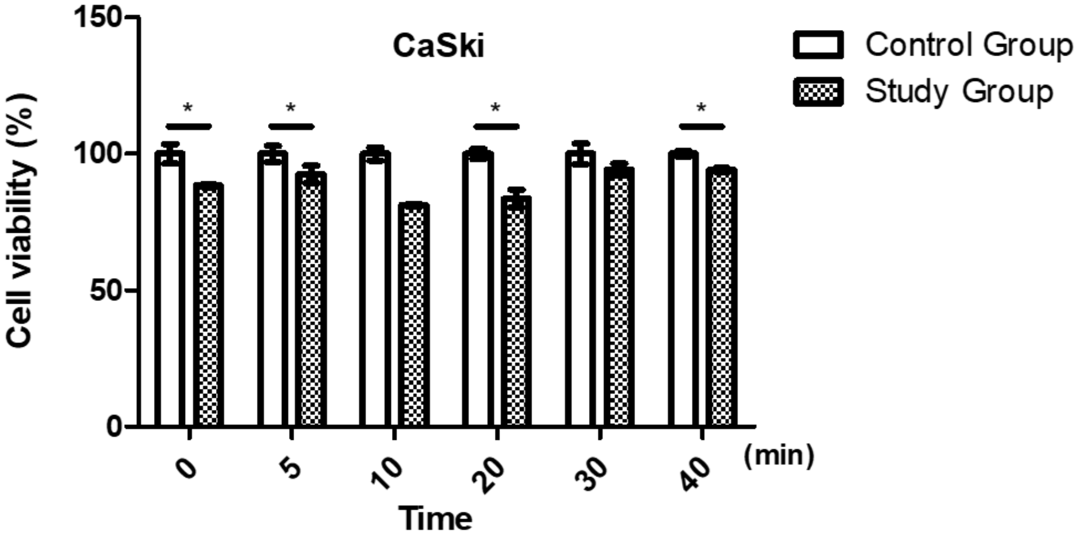

Raw Data (%)		1	2	3	Mean	SD
Water	0	95.95843	102.52	101.5215	100	3.53553
PAW		88.19454	88.01114	89.03002	88.4119	0.543107
Water	5 min	97.10585	100.0202	102.8739	100	2.884085
PAW		94.98077	93.70573	88.92937	92.53862	3.19006
Water	10 min	99.13534	102.7068	98.15789	100	2.394533
PAW		81.48496	81.48496	80.52632	81.16541	0.553475
Water	20 min	100.4544	98.00474	101.5409	100	1.811333
PAW		80.30423	83.68234	86.84315	83.6099	3.27006
Water	30 min	102.5391	101.8652	95.59564	100	3.82914
PAW		94.02314	91.9401	96.47379	94.14568	2.269331
Water	40 min	99.11455	99.69563	101.1898	100	1.070589
PAW		93.46984	93.69812	94.90177	94.02324	0.769341

Data are presented as mean±SD (n=3/group). Statistical analysis was performed using IBM SPSS Statistics (v29.0; IBM Corp., Armonk, NY, USA). Normality was assessed by the Shapiro–Wilk test and homogeneity of variance by Levene's test. Group differences were analyzed using Student's t-test or Welch's t-test, as appropriate. For non-normally distributed data, the Mann–Whitney U test was applied. Two-tailed tests were used with significance set at $^*p < 0.05$, $^{**}p < 0.01$, $^{***}p < 0.001$.
