# Supplementary material for: Efficacy and Safety of a Plasma Vaginal Cleanser (WOMEN CARE®) Using Plasma-Activated Water in Suspected Vaginitis: A Multicenter Randomized Clinical Trial
Source: Biomedicines. 2025 Dec 12;13(12):3076. doi: 10.3390/biomedicines13123076 (PMC12730254; doi:10.3390/biomedicines13123076)
Supplement: Supplementary file 1 [file biomedicines-13-03076-s001.zip › Supplement 2_Tables.pdf]

Table S2. Change of HPV Genotype status after Intervention in Control Group

Change of HPV Genotype status after Intervention in Control Group — Before (genotype)

Patients No.	16	18	31	33	35	39	45	51	52	53	56	58	59	66	67	68	69	73	82
HC-004	**																		
HC-006		***				***					***								
HC-020																*			
HC-023									*										
HC-028									**	*			*						
HC-035						***						***							
HC-042									*	**									*
HC-045						*				*				*					
HC-053						*			**										
HC-057			*																
HC-060														**					
HC-063											*								
HC-068									**										
HC-075						***				***			*	*		***			
HC-082														*					*
HC-089								***											
HC-093										**									
HC-094						**													
HC-099									*										
HC-102									**										
HC-108								**				**							
HC-110									**										
HC-129								**											
HC-132								*											
HC-133								*		*									*
ON-012						**							***	**					
ON-025	*																		
ON-028										***	**								
ON-029						**		***			**			*					
ON-055										***									
ON-060										*							*		
ON-073																		**	
ON-079		**							**	*			***					***	
ON-081			**																
ON-084	***																		
ON-085					**														
ON-088	***									**									
ON-100									**										
ON-101											**								
ON-102										**									
ON-106																		**	
ON-119									**										
ON-122						**						**							
ON-123	***					*													
ON-125		**																	
ON-133														*					
ON-137														***					
ON-138										***									
ON-139																		***	
ON-143												*							

Change of HPV Genotype status after Intervention in Control Group — After (genotype)

Patients No.	16	18	31	33	35	39	45	51	52	53	56	58	59	66	67	68	69	73	82
HC-004	x																		
HC-006		***				***					***								
HC-020										ΔΔ						*			
HC-023									**										
HC-028									**	**			*						
HC-035						***						***							
HC-042									χ	***				ΔΔ		Δ		ΔΔ	***
HC-045						**				**				**					
HC-053						χ			**										
HC-057			*																
HC-060														**					
HC-063											**								
HC-068									**										
HC-075						**				*			χ	χ		**			
HC-082								Δ			ΔΔ			**					**
HC-089								**											
HC-093										***									
HC-094						**								ΔΔ					
HC-099									χ										
HC-102									**	ΔΔ				Δ					
HC-108								***				*							
HC-110									*										
HC-129								*											
HC-132								χ											
HC-133								χ		χ									χ
ON-012						***							***	**					
ON-025	**																		
ON-028										χ	χ								
ON-029						**		***			***			χ					
ON-055										***			Δ						
ON-060										χ							χ		
ON-073																		χ	
ON-079		**							χ	**			*	Δ		Δ		***	
ON-081			*																
ON-084	***																		
ON-085					*														
ON-088	**									*									
ON-100									**										
ON-101											*								
ON-102										**									
ON-106																		**	
ON-119									*	Δ							Δ		
ON-122						χ						**							
ON-123	***					**		Δ											
ON-125		**																	
ON-133														χ					
ON-137														***					
ON-138			Δ							χ									
ON-139																		χ	
ON-143										Δ		*							

HC, Honest Clinics; ON, On Clinics; * disease severity (*–1, **–2, ***–3); X, genotype disappeared after intervention; Δ, newly acquired genotype after intervention.

Table S3. Change of HPV Genotype status after Intervention in Experimental Group

Patients No.	Change of HPV Genotype status after Intervention in Experimental Group																																					
	Before (genotype)																			After (genotype)																		
	16	18	31	33	35	39	45	51	52	53	56	58	59	66	67	68	69	73	82	16	18	31	33	35	39	45	51	52	53	56	58	59	66	67	68	69	73	82
HC-012											*		*						**											X		X						**
HC-013														***																			**					
HC-017													*																			X						
HC-018									*																			X										
HC-022													***																			***						
HC-024												*																			X		Δ					
HC-030	***								***	***				**						***								***	***				X					
HC-037												*							**												X							***
HC-041									**																			*										
HC-044						**																			X													
HC-052											*			**																X			X					
HC-054		***								**	**										*								X	X								
HC-072																*																			*			
HC-076									**																			**										
HC-087	*																			X																		
HC-096							**																			X												
HC-113	**					**														**					**													
HC-121			***			***						***										**			***	Δ					**							
HC-122																		***																			*	
HC-126				**		***										***							X		***									ΔΔ	**			
HC-128											*															Δ				**								
HC-131									**							**												**		ΔΔ					*			
HC-134			**																			**																
HC-144										***																			***									
ON-004	*								**				***							X								**				***						
ON-007																			**																			**
ON-011						*																			**													
ON-021						***																			***													
ON-039													*																			*		Δ				
ON-051										**									***										**									**
ON-053											**																			*								
ON-057																**																			***			
ON-064										*		*																	X		*							
ON-086										**			**																*			*						
ON-098									*										***									X										***
ON-104								*				*															X				X							
ON-109											**																			**			Δ					
ON-111			***																			***																
ON-131									**																			***				ΔΔΔ						
ON-134					**					*				**		*								***					***				**		*			
ON-136	*																			*																		

HC, Honest Clinics; ON, On Clinics; * disease severity (*–1, **–2, ***–3); X, genotype disappeared after intervention; Δ, newly acquired genotype after intervention.
